# A positive feedback loop promotes HIF‐1α stability through miR‐210‐mediated suppression of RUNX3 in paraquat‐induced EMT

**DOI:** 10.1111/jcmm.13264

**Published:** 2017-07-12

**Authors:** Yong Zhu, Jinfeng Wang, Xiaoxiao Meng, Hui Xie, Jiuting Tan, Xinkun Guo, Peng Han, Ruilan Wang

**Affiliations:** ^1^ Department of Critical Care Medicine Shanghai General Hospital Shanghai Jiaotong University School of Medicine Shanghai China

**Keywords:** MicroRNA‐210, hypoxia‐inducible factor‐1α, runt‐related transcription factor‐3, epithelial–mesenchymal transition, pulmonary fibrosis, paraquat poisoning

## Abstract

Irreversible pulmonary fibrosis induced by paraquat (PQ) poisoning is the major cause of death in patients with PQ poisoning. The epithelial–mesenchymal transition (EMT) is postulated to be one of the main mechanisms of pulmonary fibrosis. Here, we investigated the role of miR‐210 in PQ‐induced EMT and its relationship with hypoxia‐inducible factor‐1α (HIF‐1α). Western blotting, immunofluorescence, immunoprecipitation and other methods were used in this study. We found that miR‐210 expression was significantly increased after PQ poisoning, and it may be regulated by HIF‐1α. Overexpression of miR‐210 further increased the HIF‐1α protein level and promoted EMT. Moreover, miR‐210 knock‐down reduced the HIF‐1α protein level and decreased the degree of EMT. Runt‐related transcription factor‐3 (RUNX3), a direct target of miR‐210, was inhibited by miR‐210 in response to PQ poisoning. RUNX3 increased the hydroxylation ability of prolyl hydroxylase domain‐containing protein 2 (PHD2), a key enzyme that promotes HIF‐1α degradation. PHD2 immunoprecipitated with RUNX3 and its level changed similarly to that of RUNX3. The expression of the HIF‐1α protein was significantly reduced when RUNX3 was overexpressed. HIF‐1α protein levels were markedly increased when RUNX3 was silenced. Based on these results, a positive feedback loop may exist between miR‐210 and HIF‐1α. The mechanism may function through miR‐210‐mediated repression of RUNX3, which further decreases the hydroxylation activity of PHD2, enhances the stability of HIF‐1α, and promotes PQ‐induced EMT, aggravating the progression of pulmonary fibrosis. This study further elucidates the mechanism of PQ‐induced pulmonary fibrosis and may provide a new perspective for the future development of therapies.

## Introduction

PQ is a widely used herbicide and is highly toxic to humans and animals. It rapidly accumulates in lung tissues after PQ poisoning. Currently, no special treatments are available to prevent or cure this condition, and the mortality rate of PQ poisoning is 50–80% 1. Irreversible pulmonary fibrosis induced by PQ poisoning is the major cause of death in patients with PQ poisoning. However, methods are not available to prevent or treat PQ‐induced pulmonary fibrosis 2. Thus, studies elucidating the mechanism of PQ‐induced pulmonary fibrosis are important.

Type II alveolar epithelial cells have been shown to acquire a fibroblast‐like phenotype through EMT to promote the progression of pulmonary fibrosis 3, 4. These cells promote collagen deposition in the extracellular matrix. In addition, reduced levels of zonula occluden‐1 (ZO‐1), E‐cadherin and increased levels of α‐smooth muscle actin (α‐SMA) are indicators of the occurrence of EMT 5. The expression of the transcription factor HIF‐1α is elevated in response to low‐oxygen conditions. HIF‐1α is involved in multiple cellular functions, including blood vessel formation, cell proliferation, immunity and inflammatory responses 6, 7. As shown in our previous studies, HIF‐1α expression is significantly increased in PQ‐treated rat lung tissues, and EMT is also observed. The lung tissues are damaged, and a large number of collagen fibres are deposited. *In vitro*, PQ‐induced EMT of alveolar epithelial cells is noticeably reduced when we inhibited HIF‐1α expression. Thus, EMT, which is regulated by HIF‐1α, may be a key mechanism underlying PQ‐induced pulmonary fibrosis 8, 9. These results prompted us to identify the principal factor that regulates HIF‐1α in PQ poisoning to develop strategies to inhibit EMT.

MicroRNAs are small non‐coding RNA that regulates the expression of mRNAs at the post‐transcriptional level. Multiple microRNAs have been shown to have important roles in lung diseases, such as lung cancer, chronic obstructive pulmonary disease and pulmonary fibrosis, among others 10, 11. Among these microRNAs, microRNA‐210 (miR‐210) is closely associated with HIF‐1α and is involved in cell proliferation, differentiation and migration 12. According to the study by Oak *et al*. 13, the miR‐210 level in rapidly progressing pulmonary fibrosis is 4.5 times higher than the level in slowly progressing pulmonary fibrosis as determined by a microRNA array using patient lung biopsy samples. However, we still know very little about the role of miR‐210 in the development of pulmonary fibrosis. As shown in a recent study, miR‐210 regulates EMT in ovarian cancer cells and promotes tumour cell metastasis 14. Moreover, miR‐210 has a close relationship with HIF‐1α. Based on the results of the study by Liu, *et al*. 15, miR‐210 expression is substantially increased in connective tissue growth factor‐treated synovial fibroblasts. In addition, miR‐210 increased the stability of HIF‐1α by inhibiting glycerol‐3‐phosphate dehydrogenase 1‐like expression and promoting HIF‐1α‐dependent vascular endothelial growth factor expression. As shown in the study by Puissegur, *et al*. 16, miR‐210 inhibits degradation of HIF‐1α by repressing subunit D of the succinate dehydrogenase complex. Therefore, we hypothesize that miR‐210 may participate in PQ‐induced EMT by regulating the stability of HIF‐1α.

RUNX3 is a member of the runt‐related gene family. RUNX3 plays important roles in growth, development, immune regulation and tumourigenesis 17, 18. We identified RUNX3 as a direct target of miR‐210 using a microRNA target prediction tool (miRanda, http://www.microrna.org/microrna/getMrna.do?gene=864%26utr=2888%26organism=9606#). RUNX3 was recently shown to increase the activity of PHD2 and promote the degradation of HIF‐1α 19. Thus, we suggested that miR‐210 represses RUNX3 expression, subsequently decreasing the hydroxylation ability of PHD2, increasing the stability of HIF‐1α and promoting PQ‐induced EMT.

According to several studies, HIF‐1α directly binds to the miR‐210 promoter and regulates miR‐210 expression 20, 21, 22. Thus, a positive feedback loop between miR‐210 and HIF‐1α that aggravates PQ‐induced EMT may exist. The aim of this study was to elucidate the role of miR‐210 in PQ‐induced EMT and this may provide a potential therapeutic target to prevent PQ‐induced pulmonary fibrosis.

## Materials and methods

### Materials and reagents

PQ powder (Sigma‐Aldrich, St. Louis, MO, USA) and a 20% PQ formulation (Syngenta Crop Protection Ltd., Nantong, Jiangsu, China) were used in this study. Foetal bovine serum (FBS) and DMEM were obtained from Gibco (Grand Island, NY, USA). DMEM/F‐12 was purchased from HyClone (Logan City, UT, USA). The primary antibodies were anti‐HIF‐1α (BioWorld, St. Louis Park, MN, USA), anti‐α‐SMA, anti‐Ecadherin, anti‐OH‐HIF‐1α (P402) (Abcam, Cambridge, MA, USA), anti‐ZO‐1 (Santa Cruz Biotechnology Inc., Santa Cruz, CA, USA), anti‐RUNX3, anti‐PHD2, anti‐GAPDH and anti‐β‐actin (Cell Signaling Technology, Boston, MA, USA). Horseradish peroxidase (HRP)‐conjugated secondary antibodies, Alexa Fluor 647‐labeled goat antimouse IgG (H + L), Alexa Fluor 488‐labeled goat antimouse IgG (H + L), RIPA protein lysis buffer, PMSF, a BCA protein concentration assay kit, an SDS‐PAGE gel preparation kit, DAPI, Protein A agarose, Immunol Staining Blocking Buffer and Immunol Staining Fix Solution were purchased from Beyotime (Shanghai, China). PVDF membranes (Bio‐Rad, Richmond, CA, USA) and a highly sensitive enhanced chemiluminescence (ECL) agent were obtained from Thermo Fisher Scientific (Waltham, MA, USA), and TRIzol and Lipofectamine™ 2000 were obtained from Invitrogen (Grand Island, NY, USA). The SYBR^®^ Premix Ex Taq™ Kit and Prime Script™ RT Master Mix Kit were obtained from TaKaRa (Dalian, Liaoning, China).

### Animal experiments

Sprague Dawley (SD) rats were purchased from the Chinese Academy of Sciences experiment centre in Shanghai. Sixty‐six healthy male SD rats were randomly divided into a control group (*n* = 6) and a PQ group (*n* = 60). The PQ group was randomly and evenly divided into six subgroups according to the different times of examination (2, 6, 12, 24, 48 and 72 hrs) after PQ treatment (*n* = 10). The PQ group was treated with an intragastric infusion of 20% PQ solution (50 mg/kg) and the control group received the same volume of saline. All animal protocols were approved by the Ethics Committee of Shanghai General Hospital. Detailed descriptions of the experiments are provided in our previous study 9.

### Cell culture and PQ treatment

Human lung adenocarcinoma epithelial cells (A549) and rat alveolar type II cells (RLE‐6TN) were obtained from the American Type Culture Collection (ATCC, Rockville, MD, USA). Briefly, A549 cells were grown in DMEM supplemented with 10% FBS (Gibco) and 1% antibiotics (100 U/ml penicillin, 0.1 mg/ml streptomycin). RLE‐6TN cells were cultured in DMEM/F‐12 supplemented with 10% FBS and 1% antibiotics. Both cells were grown at 37°C in a 5% carbon dioxide incubator. The final PQ concentrations used to treat A549 cells was 800 μm/l and RLE‐6TN cells were treated with 160 μm/l PQ; both cells were treated for 24 hrs. The details are described in our earlier study 9.

### Real‐time quantitative PCR (qRT‐PCR) analysis of mRNA expression

Total RNA was extracted from cells using TRIzol reagent. The concentration of the total RNA was determined using an ultraviolet spectrophotometer. Reverse transcription was conducted using a Prime Script™ RT Master Mix Kit according to the manufacturer's instruction. qRT‐PCR was performed using a SYBR^®^ Premix Ex Taq™ Kit and an ABI ViiA™ 7 System. The specific primers for β‐actin, HIF‐1α and RUNX3 were generated by BioTNT (Shanghai, China) and are shown inTable S1. All samples were assayed in triplicate, and the values were normalized to β‐actin.

### qRT‐PCR analysis of miR‐210

Total RNA was isolated from rat lung tissues and cells with TRIzol. The cDNA was synthesized by stem‐loop qRT‐PCR using the BioTNT microRNA qRT‐PCR SYBR Green Detection Kit (Part A). qRT‐PCR amplification was performed as described above. Human U6B and rat U6B levels were used to normalize the expression of target microRNA. The primers used for qRT‐PCR were purchased from BioTNT. All samples were assayed in triplicate.

### Western blotting

Total proteins were extracted from the cells in each group using RIPA buffer. The protein concentrations were determined with a BCA protein assay kit. The total protein samples were separated using 10% SDS‐PAGE, transferred to a PVDF membrane, blocked with 5% non‐fat milk in Tris‐buffered saline with Tween 20 (TBST), and incubated with primary antibodies against HIF‐1α (1:500), α‐SMA (1:1000), E‐cadherin (1:500), ZO‐1 (1:500), RUNX3 (1:1000), PHD2 (1:1000), OH‐HIF‐1α (P402) (1:500), GAPDH (1:500) and β‐actin (1:3000) overnight at 4°C. The secondary antibodies, HRP‐conjugated goat anti‐rabbit IgG (1:2000) and goat antimouse IgG (1:2000), were incubated with the membranes for 1.5 hrs at room temperature. After the membranes were washed in TBST, the bands were visualized with the ECL detection system according to the manufacturer's protocol.

### Transient transfection

The HIF‐1α siRNA, miR‐210 mimics, miR‐210 inhibitors, RUNX3 siRNA and scrambled control sequences were purchased from GenePharma (Shanghai, China). RUNX3 plasmid was generated by GeneChem (Shanghai, China) using the GV208 vector. Cells were transiently transfected with the HIF‐1α siRNA, miR‐210 mimics, miR‐210 inhibitors, RUNX3 siRNA, RUNX3 plasmids or scrambled control sequences using Lipofectamine 2000 according to the manufacturer's protocol, with slight modifications. Specifically, both cells were cultured in six‐well culture plates 24 hrs prior to transfection. Then, 4 μl of Lipofectamine 2000 were incubated with 100 pmol of siRNA/mimics/inhibitors (2 μg plasmids) or negative control sequences in 500 μl of Opti‐MEM for 20 min. at room temperature. Cells were transfected by replacing the medium with 2 ml of Opti‐MEM containing the siRNA/mimics/inhibitors (plasmids) or negative control sequences and Lipofectamine 2000, and incubated at 37°C in a humidified atmosphere of 5% CO_2_ for 6 hrs. Then, the Opti‐MEM was replaced with 2 ml of fresh culture medium. 24 or 48 hrs, the cells were incubated with PQ for 24 hrs, collected, and real‐time PCR and Western blotting assays were performed.

### Immunofluorescence

The A549 cells were seeded in the confocal dishes 24 hrs before treatment. Then, the cells were transfected with miR‐210 mimics or inhibitors as described above. After a 24 hrs treatment with PQ, the cells were washed with PBS three times, fixed with Immunol Staining Fix Solution for 10 min., and blocked with Immunol Staining Blocking Buffer for 1 hr at room temperature. The cells were incubated with a primary antibody (E‐cadherin, 1:50; α‐SMA, 1:50) overnight at 4°C. The cells were then incubated with Alexa Fluor 488‐labeled goat antimouse IgG (E‐cadherin) or Alexa Fluor 647‐labeled goat antimouse IgG (a‐SMA) secondary antibodies for 1.5 hr at room temperature after three washes with TBST. Nuclei were stained with DAPI for 5 min. Finally, the cells were observed under a laser confocal microscope (Leica TCS SP8; Leica, Wetzlar, Germany).

### Immunoprecipitation

After transfection with miR‐210 mimics or inhibitors and treatment with PQ, A549 and RLE‐6TN cells were lysed with RIPA lysis buffer. The lysates were incubated on ice for at least 30 min., centrifuged at 12,000 g for 15 min. and the supernatants were collected. The protein concentrations of the supernatants were determined using the BCA protein concentration assay kit. Then, the solution was incubated with the RUNX3 antibody overnight at 4°C and mixed with Protein A agarose (40 μl) for 3 hrs at 4°C. Subsequently, the immunoprecipitation solution was centrifuged at 2500 g for 5 min., and the pellet was washed five times with PBS. The complex was heated at 100°C for 10 min. with 1× loading buffer and then subjected to Western blotting analysis.

### Luciferase assay

The 293T cells were incubated at 37°C in a humidified atmosphere of 5% CO_2_, and 1.5 × 10^4^ cells were grown in 96‐well plates for 24 hrs with 100 μl of medium. First, the miR‐210 mimics or non‐target controls (NC) were diluted with 10 μl of Opti‐MEM, and the RUNX3 3ˈUTR wild‐type vector or mutant vector was diluted with 15 μl Opti‐MEM. Lipofectamine™ 2000 (0.25 μl) was added to 25 μl Opti‐MEM medium for 5 min. Then, the three compounds were mixed in a final volume of 50 μl and incubated for 20 min. The mixture was pipetted into each well after 50 μl of medium were removed. Each group consisted of three wells. After 6 hrs, 100 μl fresh medium were added to the wells. Then, 35 μl of fresh medium and 35 μl of luciferase substrate were added to the wells. Finally, the reactions were stopped with 30 μl of stop reagent and incubated for 10 min. Luciferase activity was measured using the Dual‐Glo^®^ Luciferase Assay System (Promega, Madison, WI, USA) according to the manufacturer's instructions.

### Statistical analysis

All data were analysed using SPSS (version 16.0; Chicago, IL, USA). Three independent experiments were performed. Data are expressed as means ± standard deviations. A *t*‐test was used for comparisons between two groups. Statistical significance was set at *P *<* *0.05.

## Results

### miR‐210 is expressed at high levels *in vivo* and *in vitro*, and is regulated by HIF‐1α

**Figure 1:**
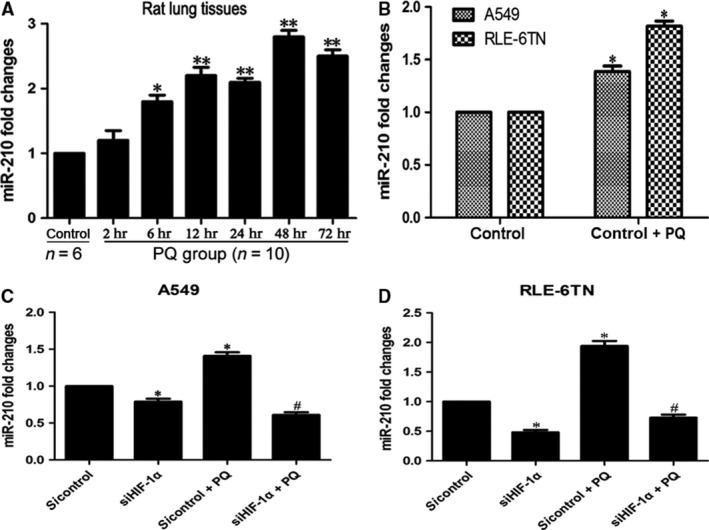
HIF‐1α modulates miR‐210 expression in PQ poisoning. (**A**) The levels of miR‐210 in rat lung tissues were detected by qRT‐PCR. U6B served as a loading control. *P <* *0.05 compared with control group, **P <* *0.01 compared with control group. (**B**) The miR‐210 levels were measured by qRT‐PCR in A549 and RLE‐6TN cells after PQ treatment. U6B served as a loading control. *P <* *0.05 compared with control group. (**C** and **D**) The expression of miR‐210 in both cells after HIF‐1α silencing. *P <* *0.05 compared with sicontrol group; #*P *<* *0.05 compared with sicontrol + PQ group.

We measured miR‐210 expression *in vivo* and *in vitro* to investigate its role in PQ‐induced EMT. According to the qRT‐PCR analysis, miR‐210 expression was up‐regulated at 6 hrs and increased with time in the PQ‐treated rat lung tissues (Fig. 1A). The levels of miR‐210 were also markedly increased in both cells after PQ treatment *in vitro* (Fig. 1B). The expression of miR‐210 was substantially decreased when HIF‐1α expression was silenced in the A549 (Fig. 1C) and RLE‐6TN cells (Fig. 1D). The morphology of HIF‐1α inhibited cells at 48 and 72 hrs (without PQ) did not change notably (Fig. S1).

### miR‐210 may regulate PQ‐induced EMT through HIF‐1α

**Figure 2:**
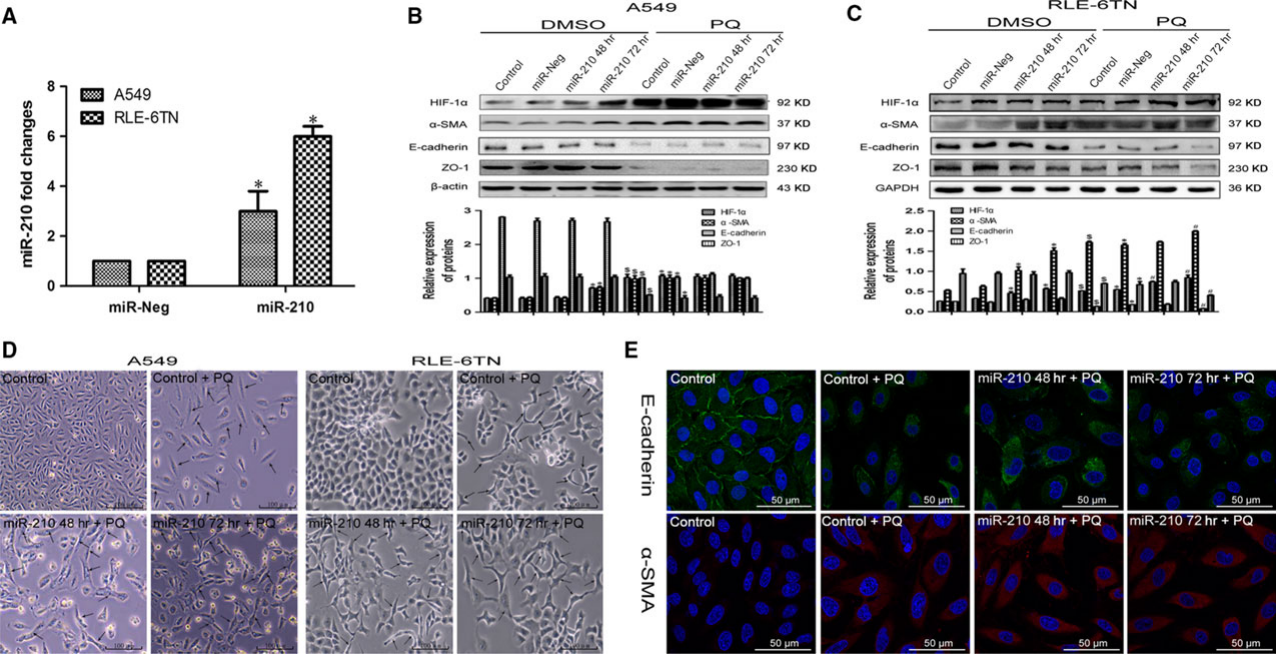
Overexpression of miR‐210 affects the expression of HIF‐1α and EMT‐related proteins. (**A**) A549 and RLE‐6TN cells were transfected with miR‐210 mimics. The miR‐210 levels were determined by qRT‐PCR, and U6B was used as a loading control. *P <* *0.05 compared with miR‐Neg group. (**B** and **C**) Levels of the HIF‐1α, α‐SMA, E‐cadherin and ZO‐1 protein in both miR‐210‐overexpressing cells were detected by Western blotting analysis. GAPDH and β‐actin served as the loading controls. $*P *<* *0.05 compared with control group; *P <* *0.05 compared with miR‐Neg group; #*P *<* *0.05 compared with miR‐Neg + PQ group. (**D**) Morphological changes were observed in both cells using phase contrast microscopy. Scale bar: 100 μm. Arrows indicated to be fusiform. (**E**) Changes in E‐cadherin and α‐SMA expression in miR‐210‐overexpressing A549 cells were detected using immunofluorescence. Scale bar: 50 μm.

Next, we investigated the effect of miR‐210 on HIF‐1α expression and EMT‐related proteins. Both cells were transfected with miR‐210 mimics. According to the qRT‐PCR analysis, miR‐210 expression was significantly increased compared with that in the miR‐Neg group (Fig. 2A). In A549 cells, HIF‐1α and α‐SMA levels were significantly increased in the miR‐210 overexpression group at 72 hrs compared with those in the miR‐Neg group. Levels of the ZO‐1 and E‐cadherin proteins were not significantly changed (Fig. 2B). However, in the RLE‐6TN cells, HIF‐1α and α‐SMA expressions were further increased in response to miR‐210 overexpression compared with the expression in the miR‐Neg and miR‐Neg + PQ groups. ZO‐1 and E‐cadherin levels were reduced in the miR‐210 72 hrs + PQ group (Fig. 2C). Using phase contrast microscopy, we observed fusiform cells in both cells after overexpressing miR‐210 compared with the control + PQ group (Fig. 2D). Based on the immunofluorescence results, E‐cadherin expression was significantly decreased and α‐SMA expression was markedly increased in miR‐210 overexpressing A549 cells (Fig. 2E).

**Figure 3:**
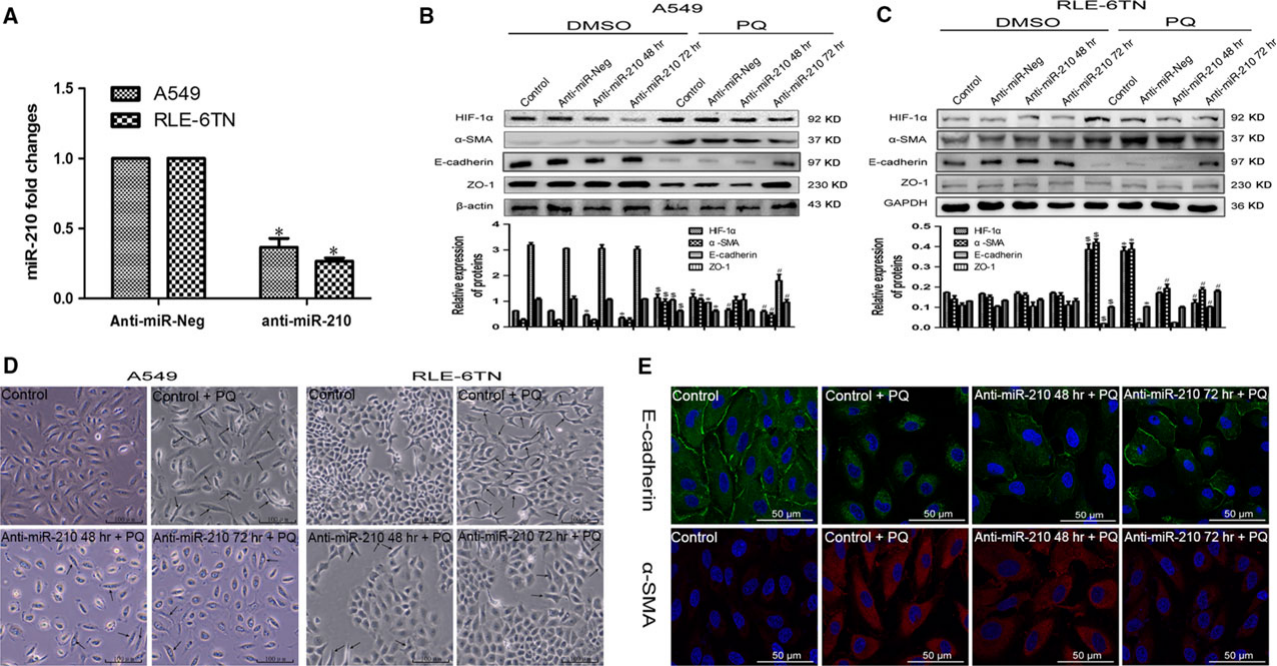
Silencing of miR‐210 affects the expression of HIF‐1α and EMT‐related proteins. (**A**) A549 and RLE‐6TN cells were transfected with miR‐210 inhibitors. The miR‐210 levels were determined by qRT‐PCR. U6B was used as a loading control. *P <* *0.05 compared with anti‐miR‐Neg group. (**B** and **C**) Levels of HIF‐1α, α‐SMA, E‐cadherin and ZO‐1 protein in both miR‐210‐silenced cells were detected by Western blotting analysis. GAPDH and β‐actin served as loading controls. $*P *<* *0.05 compared with control group; *P <* *0.05 compared with anti‐miR‐Neg group; #*P *<* *0.05 compared with anti‐miR‐Neg + PQ group. (**D**) Morphological changes were observed in both cells using phase contrast microscopy. Scale bar: 100 μm. Arrows indicated to be fusiform. (**E**) Changes in E‐cadherin and α‐SMA expression in miR‐210‐silenced A549 cells were detected using immunofluorescence. Scale bar: 50 μm.

Then, we inhibited miR‐210 expression by transfecting the cells with miR‐210 inhibitors. Based on the qRT‐PCR data, miR‐210 expression was successfully inhibited in both cells (Fig. 3A). In A549 cells, HIF‐1α expression was significantly decreased in the miR‐210 inhibitor group compared with that in the anti‐miR‐Neg and anti‐miR‐Neg + PQ groups. The α‐SMA level was significantly decreased in the anti‐miR‐210 72 hrs + PQ group. ZO‐1 and E‐cadherin expressions increased (Fig. 3B). In RLE‐6TN cells, HIF‐1α and α‐SMA expressions were also markedly reduced after inhibition of miR‐210 and the PQ treatment. ZO‐1 and E‐cadherin expressions were elevated in the miR‐210 inhibitor group after 72 hrs (Fig. 3C). The degree of morphological changes in both cells was reduced after miR‐210 expression was silenced (Fig. 3D). Based on the immunofluorescence results, E‐cadherin expression was significantly increased and α‐SMA expression was markedly reduced at 72 hrs after miR‐210 expression was silenced in A549 cells (Fig. 3E).

### miR‐210 may influence the prolyl hydroxylation of HIF‐1α by regulating PHD2 activity

**Figure 4:**
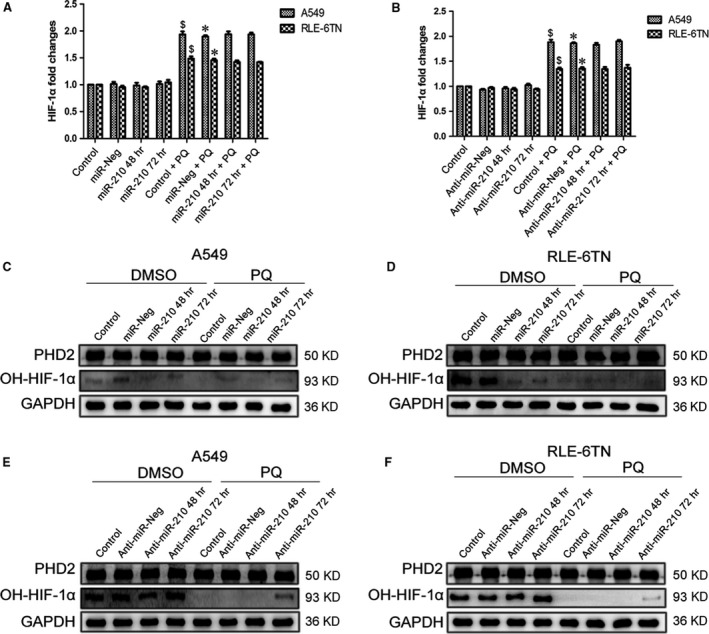
miR‐210 did not affect the expression of the HIF‐1α and PHD2 proteins. (**A**) A549 and RLE‐6TN cells were transfected with miR‐210 mimics. HIF‐1α mRNA levels were determined by qRT‐PCR, and β‐actin was used as a loading control. $*P *<* *0.05 compared with control group; *P <* *0.05 compared with miR‐Neg group.(**B**) Both cells were transfected with miR‐210 inhibitors. HIF‐1α mRNA levels were detected by qRT‐PCR. β‐actin was used as a loading control. $*P *<* *0.05 compared with control group; *P <* *0.05 compared with anti‐miR‐Neg group. (**C** and **D**) Levels of PHD2 and OH‐HIF‐1α protein in both miR‐210‐overexpressing cells were detected by Western blotting analysis. GAPDH served as the loading control. (**E** and **F**) Levels of PHD2 and OH‐HIF‐1α protein in both miR‐210‐silenced cells were detected by Western blotting analysis. GAPDH served as the loading control.

We first measured the change in the expression of HIF‐1α mRNA to investigate the underlying mechanism by which miR‐210 regulated HIF‐1α. We overexpressed or inhibited miR‐210 in A549 and RLE‐6TN cells, as described above. Based on the results of the qRT‐PCR analysis, the HIF‐1α mRNA levels were not significantly different in the DMSO group and the PQ group after increasing (Fig. 4A) or repressing (Fig. 4B) miR‐210 expression. Although the level of PHD2 protein was not significantly altered either cell when we increased miR‐210 expression, the OH‐HIF‐1α levels were significantly decreased (Fig. 4C and D). The expression of OH‐HIF‐1α was markedly increased following the down‐regulation of miR‐210 (Fig. 4E and F). Based on the Western blotting results, the levels of OH‐HIF‐1α were significantly decreased by the PQ treatment.

### miR‐210 regulates PHD2 activity by altering RUNX3 expression

**Figure 5:**
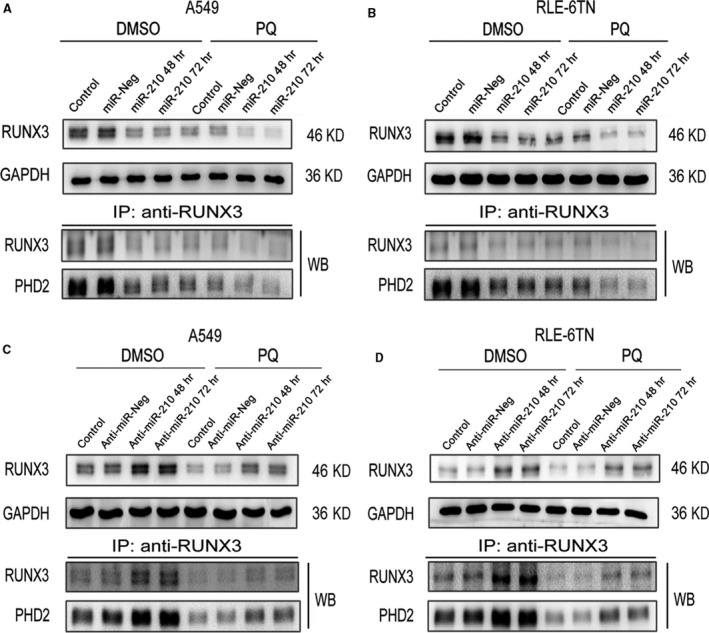
RUNX3 protein levels were negatively correlated with miR‐210 expression. (**A** and **B**) Levels of the RUNX3 protein in both miR‐210‐overexpressing A549 and RLE‐6TN cells were detected by Western blotting analysis. GAPDH served as the loading control. The cell extracts were immunoprecipitated using an anti‐RUNX3 antibody, and the precipitated proteins were analysed by Western blotting with anti‐RUNX3 and anti‐PHD2 antibodies. (**C** and **D**) Levels of the RUNX3 protein in both miR‐210‐silenced cells were detected by Western blotting analysis. GAPDH served as the loading control. The cell extracts were immunoprecipitated using an anti‐RUNX3 antibody, and the precipitated proteins were analysed by Western blotting with anti‐RUNX3 and anti‐PHD2 antibodies.

RUNX3 was predicted to be a target of miR‐210. First, RUNX3 expression was significantly decreased by the PQ treatment. Moreover, the levels of RUNX3 protein were drastically reduced in miR‐210‐overexpressing cells in the DMSO and PQ groups at 48 and 72 hrs. PHD2 immunoprecipitated with RUNX3, and its levels changed similarly to those of RUNX3 (Fig. 5A and B). RUNX3 expression was substantially increased in both miR‐210‐silenced cells at 48 and 72 hrs (Fig. 5C and D). Moreover, PHD2 levels were also increased similar to the RUNX3 levels.

### miR‐210 directly regulates RUNX3 through its 3′UTR

**Figure 6:**
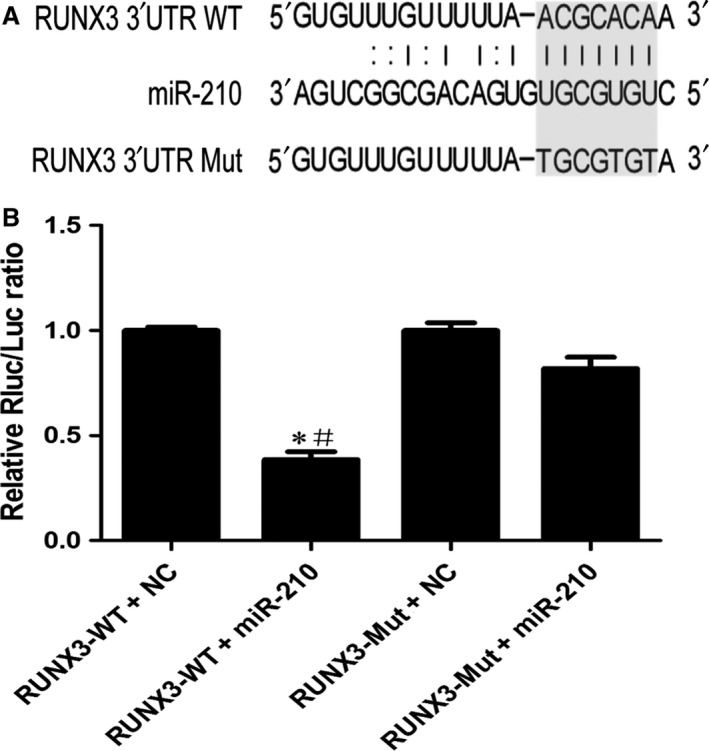
RUNX3 is a direct target of miR‐210. (**A**) Diagram of miR‐210 binding site in RUNX3 3′UTR. RUNX3 3′UTR mutant was constructed to evaluate miR‐210 binding. (**B**) The wild‐type or mutant RUNX3 3′UTR vector was transfected into 293T cells with miR‐210 mimics or NC (non‐target controls) using Lipofectamine™ 2000. Luciferase activity was measured using the Dual‐Glo^®^ Luciferase Assay System (Promega). *P <* *0.05 compared with RUNX3‐WT + NC group, #*P *<* *0.05 compared with RUNX3‐Mut + miR‐210 group.

Next, we confirmed that RUNX3 is a direct target of miR‐210 using luciferase reporter assays. Figure 6A shows the proposed microRNA binding site in 3′UTR of RUNX3 gene as well as the mutant 3′UTR that was generated in this study. As shown in Figure 6B, the luciferase activity was significantly decreased in the RUNX3‐WT + miR‐210 group compared with that in the RUNX3‐WT + NC and RUNX3‐Mut + miR‐210 groups. Based on these results, RUNX3 is a direct target of miR‐210.

### RUNX3 may regulate HIF‐1α by influencing PHD2 activity

**Figure 7:**
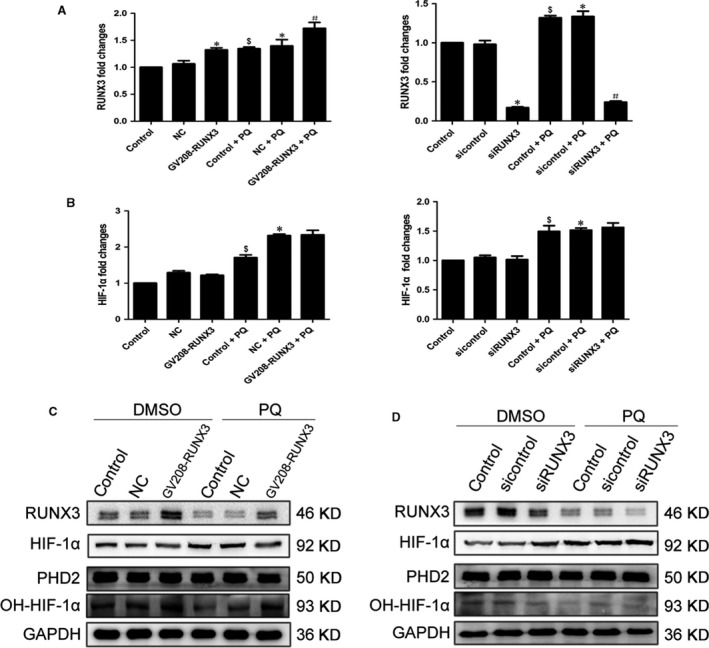
RUNX3 may regulate HIF‐1α by influencing PHD2 activity. (**A**) We overexpressed or silenced RUNX3 by transfecting A549 cells with RUNX3 plasmid or siRNA for 48 hrs. The expression of the RUNX3 mRNA was assessed using qRT‐PCR analysis. $*P *<* *0.05 compared with control group; *P <* *0.05 compared with NC or sicontrol group; #*P *<* *0.05 compared with NC + PQ or sicontrol + PQ group. NC indicates the negative control group. (**B**) HIF‐1α mRNA changes in A549 cells after increasing or silencing RUNX3. $*P *<* *0.05 compared with control group; *P <* *0.05 compared with NC or sicontrol group. NC indicates the negative control group. (**C** and **D**) Levels of the RUNX3, HIF‐1α, PHD2 and OH‐HIF‐1α proteins in RUNX3‐overexpressing or ‐silenced were detected by Western blotting analysis. GAPDH served as a loading control.

Next, we evaluated the effect of RUNX3 on HIF‐1α in PQ poisoning. RUNX3 was significantly overexpressed or reduced in A549 cells following transfection with the RUNX3 plasmid or siRNA, respectively, for 48 hrs (Fig. 7A). However, the HIF‐1α mRNA level was not significantly changed in the DMSO and PQ groups after RUNX3 was overexpressed or silenced (Fig. 7B). The levels of RUNX3 and OH‐HIF‐1α proteins were markedly increased, and the levels of HIF‐1α protein were decreased in both groups in response to RUNX3 overexpression (Fig. 7C). As shown in the Western blot results, levels of the RUNX3 and OH‐HIF‐1α protein were substantially decreased, and the level of the HIF‐1α protein increased in both groups in response to RUNX3 inhibition (Fig. 7D). Nevertheless, the levels of PHD2 protein were not changed (Fig. 7C and D).

## Discussion

The lung is the main target organ of PQ poisoning. The concentration of PQ in the lung is approximately 6–10 times higher than the concentration in the blood 2. Irreversible pulmonary fibrosis leading to respiratory failure is still the main cause of PQ poisoning‐induced death 23. Oxidative stress, endoplasmic reticulum stress, inflammation and alveolar epithelial cell apoptosis are possible mechanisms of PQ‐induced pulmonary fibrosis 24, 25, 26, 27. Recent years, many studies have confirmed the role of EMT in the development of pulmonary fibrosis. Epithelial cells acquire mesenchymal features and exhibit increased deposition of the extracellular matrix 5, 28. As shown in our previous study, EMT also participates in PQ‐induced pulmonary fibrosis and may be regulated by HIF‐1α 9. In this study, we found that miR‐210 regulated PQ‐induced EMT by regulating HIF‐1α expression through inhibiting RUNX3 expression and decreasing the hydroxylation of HIF‐1α by PHD2. These results may provide a new perspective for future development of therapies.

MicroRNAs are endogenous small non‐coding RNA molecules that post‐transcriptionally regulate gene expression in diverse biological and pathological processes. MicroRNAs are involved in regulating EMT in pulmonary fibrosis 29. Here, miR‐210 expression was markedly increased after PQ poisoning *in vivo* and *in vitro*. Based on these results, miR‐210 may play a role in the development of PQ‐induced pulmonary fibrosis. A direct regulatory role of HIF‐1α in the transcription of miR‐210 has recently been reported 20, 30, 31. The silencing of HIF‐1α expression also reduced miR‐210 expression. Thus, miR‐210 expression was regulated by HIF‐1α in PQ‐treated alveolar epithelial cells. However, several researchers discovered that miR‐210 also regulates HIF‐1α expression 16, 32. We overexpressed miR‐210 in A549 and RLE‐6TN cells, and changes in HIF‐1α expression were observed. HIF‐1α expression decreased when miR‐210 expression was reduced. Based on our findings, the relationship between miR‐210 and HIF‐1α expression was not unidirectional; miR‐210 also regulated the expression of HIF‐1α in response to PQ poisoning.

We measured EMT markers after overexpressing or inhibiting miR‐210 expression in both cells to further explore the role of miR‐210 in PQ‐induced EMT. Overexpression of miR‐210 promoted EMT and miR‐210 inhibition alleviated EMT. Based on these results, miR‐210 affected PQ‐induced EMT, possibly *via* the relationship between miR‐210 and HIF‐1α. Thus, miR‐210 may modulate PQ‐induced EMT by targeting HIF‐1α.

We assessed changes in HIF‐1α mRNA levels after altering miR‐210 expression to further elucidate the mechanism by which miR‐210 regulates HIF‐1α. However, the HIF‐1α mRNA level was not significantly altered in either the A549 or RLE‐6TN cells, indicating that miR‐210 had no effect on HIF‐1α transcription. Then, we suggested miR‐210 may affect the degradation of the HIF‐1α protein. HIF‐1α is constitutively expressed under normoxic conditions, but post‐translationally modified by a class of 2‐oxoglutarate‐dependent and Fe2+‐dependent prolyl hydroxylases (PHDs) at prolines 402 and 564; the modified protein is degraded after ubiquitination by von Hippel Lindau (pVHL), a tumour suppressor 33, 34. HIF‐1α is also regulated by transactivational inhibition of asparagine 803, which is hydroxylated by Factor Inhibiting HIF (FIH) 35. The PHDs family include PHD1, PHD2 and PHD3. PHD2 is believed to be a key oxygen sensor that hydroxylates HIF‐1α and promotes its degradation 36, 37. OH‐HIF‐1α levels were markedly decreased in PQ group. Thus, the increased HIF‐1α level was caused by the reduced degradation of HIF‐1α after the PQ treatment. OH‐HIF‐1α levels were also negatively correlated with the miR‐210 levels. However, the level of PHD2 protein was not significantly changed. Based on these data, miR‐210 may regulate HIF‐1α levels by regulating PHD2 activity and subsequently influencing the prolyl hydroxylation of HIF‐1α.

**Figure 8:**
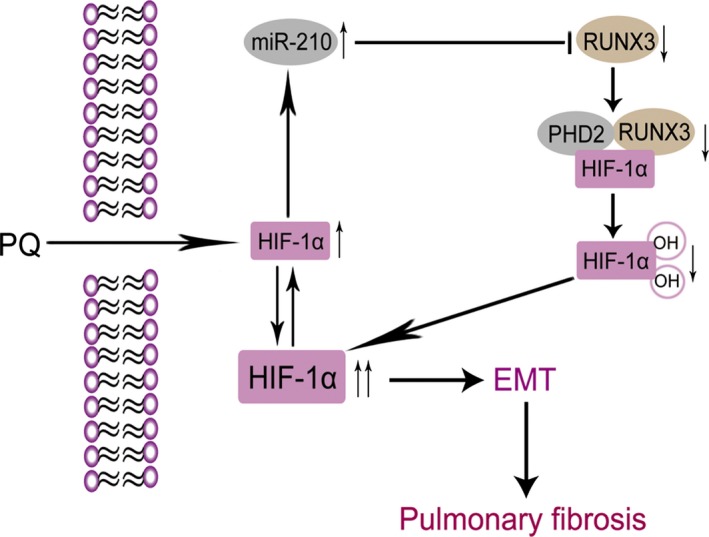
Potential pathway by which miR‐210 regulates PQ‐induced EMT. A positive feedback loop between miR‐210 and HIF‐1α was observed. In PQ poisoning, HIF‐1α overexpression increases miR‐210 expression. Then, miR‐210 represses RUNX3 expression, decreases hydroxylation activity of PHD2, increases HIF‐1α stability, participates in PQ‐induced EMT and aggravates the progression of pulmonary fibrosis.

RUNX3 is a tumour suppressor gene that functions in the early stage, and it is involved in immunity, inflammation, apoptosis and development 17, 38. As shown in the study by Lee *et al*. 19, RUNX3 could decrease the half‐life of HIF‐1α, as well as its nuclear localization under hypoxia. Moreover, RUNX3 directly interacts with the C‐terminal activation domain of HIF‐1α and PHD2, promoting their interaction. Subsequently, it induces hydroxylation at prolines 402 and 564 in the oxygen dependent degradation domain, promoting the degradation of HIF‐1α, suggesting that RUNX3 is essential for PHD2‐mediated binding and hydroxylation of HIF‐1α. The decreased levels of RUNX3 in the PQ group showed that RUNX3 may participate in the PQ‐induced increase in the HIF‐1α levels by reducing the hydroxylation activity of PHD2 towards HIF‐1α. We confirmed that RUNX3 is a direct target of miR‐210. RUNX3 protein levels were negatively correlated with miR‐210 expression in PQ‐induced EMT. In addition, RUNX3 immunoprecipitated with PHD2. The PHD2 level changed similarly to the RUNX3 level. Based on these levels, RUNX3 may affect the hydroxylation activity of PHD2 towards HIF‐1α. Moreover, the levels of the HIF‐1α protein were dramatically reduced when we overexpressed RUNX3. The levels of the HIF‐1α protein were markedly increased when we silenced RUNX3. Thus, miR‐210 may function by repressing RUNX3 expression, resulting in the decreased hydroxylation activity of PHD2, enhanced stability of HIF‐1α, increased levels of the HIF‐1α protein, and aggravation of PQ‐induced EMT and pulmonary fibrosis (Fig. 8).

In conclusion, we found that there was a positive feedback loop between miR‐210 and HIF‐1α in PQ‐induced EMT. Each of the two molecules promoted the activity of the other to aggravate PQ‐induced EMT and further promote pulmonary fibrosis. This study may provide a potential therapeutic target for PQ poisoning and help to elucidate other pathophysiological mechanisms involved in pulmonary fibrosis development arising from many other causes.

## Conflict of interest

The authors declare that there are no conflicts of interest.

## Supporting information


**Figure S1** The morphology of HIF‐1α inhibiyed cells at 48 and 72 hrs (without paraquat).

**Table S1** The primer sequences used in qRT‐PCR.
