# Supplementary figures and images for: A positive feedback loop promotes HIF‐1α stability through miR‐210‐mediated suppression of RUNX3 in paraquat‐induced EMT
Source: J Cell Mol Med. 2017 Jul 12;21(12):3529–39. doi: 10.1111/jcmm.13264 (PMC5706527; doi:10.1111/jcmm.13264)

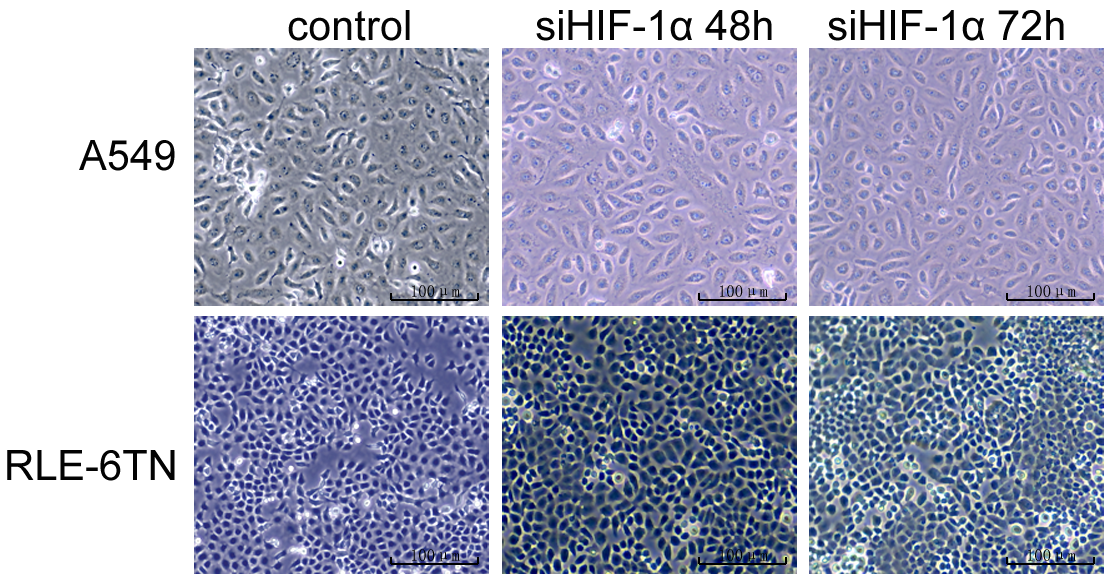

Supplement: Supplementary file 1 — Figure S1 The morphology of HIF‐1α inhibiyed cells at 48 and 72 hrs (without paraquat). [file JCMM-21-3529-s001.tif]
