# Supplementary material for: A positive feedback loop promotes HIF‐1α stability through miR‐210‐mediated suppression of RUNX3 in paraquat‐induced EMT
Source: J Cell Mol Med. 2017 Jul 12;21(12):3529–39. doi: 10.1111/jcmm.13264 (PMC5706527; doi:10.1111/jcmm.13264)
Supplement: Supplementary file 2 — Table S1 The primer sequences used in qRT‐PCR. [file JCMM-21-3529-s002.doc]

**Table**

Table S1 The primer sequences used in qRT-PCR.

Species Gene Primer sequence (Forward primer; Reverse primer)

Homo HIF-1α GTCTGAGGGGACAGGAGGAT; CTCCTCAGGTGGCTTGTCAG

sapiens RUNX3 CTCAACGCATCCACTCTCTG; CCCTCCTGTTCTCTCCACAA

β-actin CTGGAACGGTGAAGGTGACA;

AGGGACTTCCTGTAACAATGCA

Rattus HIF-1α AAGTCTAGGGATGCAGCACG; AGATGGGAGCTCACGTTGTG

Norvegicus RUNX3 ACTTCCTCTGCTCCGTGCT; GTCACCACCGTTCCATCAGG

β-actin AGGATGCAGAAGGAGATTACTGC;

AAAACGCAGCTCAGTAACAGTGC

These primer sequences were used in qRT-PCR for HIF-1α, RUNX3 and β-actin.
